# Assessment of mesh shrinkage using fibroblast-populated collagen matrices: a proof of concept for in vitro hernia mesh testing

**DOI:** 10.1007/s10029-023-02941-6

**Published:** 2024-01-05

**Authors:** R. Khader, T. Whitehead-Clarke, V. Mudera, A. Kureshi

**Affiliations:** Centre for 3D Models of Health and Disease, Division of Surgery & Interventional Science, University College London, London, UK

**Keywords:** Hernia, Mesh, In vitro, Testing, Materials

## Abstract

**Purpose:**

This study uses free-floating contractile fibroblast-populated collagen matrices (FPCMs) to test the shrinkage of different hernia mesh products. We hope to present this model as a proof of concept for the development of in vitro hernia mesh testing—a novel technology with interesting potential.

**Methods:**

FPCMs were formed by seeding Human Dermal Fibroblasts into collagen gels. FPCMs were seeded with three different cell densities and cast at a volume of 500 μl into 24-well plates. Five different mesh products were embedded within the collagen constructs. Gels were left to float freely within culture media and contract over 5 days. Photographs were taken daily and the area of the collagen gel and mesh were measured. Media samples were taken at days 2 and 4 for the purposes of measuring MMP-9 release. After 5 days, dehydrated FPCMs were also examined under light and fluorescence microscopy to assess cell morphology.

**Results:**

Two mesh products—the mosquito net and large pore lightweight mesh were found to shrink notably more than others. This pattern persisted across all three cell densities. There were no appreciable differences observed in MMP-9 release between products.

**Conclusions:**

This study has successfully demonstrated that commercial mesh products can be successfully integrated into free-floating contractile FPCMs. Not only this, but FPCMs are capable of applying a contractile force upon those mesh products—eliciting different levels of contraction between mesh products. Such findings demonstrate this technique as a useful proof of concept for future development of in vitro hernia mesh testing.

## Introduction

It has been estimated that around one million meshes are used for hernia repair each year [1]. In recent decades, mesh development has advanced exponentially as industry continues to create new biomaterial designs with beneficial properties. Currently there are over one hundred mesh variations on the market [2], including conventional synthetic meshes as well as newer biological products. More recently, industry has continued this innovation by developing new absorbable “biosynthetic” meshes [3].

Arguably the most common polymer used for mesh production is polypropylene (PP) due to its purported flexibility, strength, and tissue response [2]. PP is also well-established in the scientific literature—being originally described by Usher in 1959 [4]. Other common polymers include polyester, expanded polytetrofluroethane (ePTFE) and polyvinylidene (PVDF). As well as different materials, devices can be manufactured with a wide array of different characteristics. Mesh properties such as weight, thickness, filament size and pore size can all vary significantly between products. Arguably, the two most relevant of these characteristics are mesh weight and pore size. The weight of mesh is dependent upon both the amount of material used and weight of the polymer itself [5]. Heavyweight meshes typically have smaller pores and are made of thick polymers that provide a higher tensile strength. These meshes commonly weigh up to 120 g/m^2^. Contrastingly, lightweight meshes often consist of lighter, thinner filaments and contain larger pores [6]. Lightweight meshes generally weigh less than 70 g/m^2^ [7]. These meshes tend to contain less material, and, therefore, produce a milder foreign body reaction [6]. For the purposes of tissue integration, it has been suggested that pore size, and even pore shape, plays a significant role [8]. Some have suggested that meshes with a large pore size allow infiltration of fibroblasts and collagen fibres that initiate the formation of connective tissue, leading to better integration [6].

Although meshes have been proven to reduce hernia recurrence, devices have faced scrutiny in recent years due to complications, including infection [9], migration [10] and erosion [11]. As well as these localised phenomena, some authors describe mesh devices causing a systemic reaction known as ASIA syndrome [12]. Scrutiny over mesh devices has lead EU medical devices regulators to alter the requirements for new mesh products entering the market [13], increasing the need for human testing. The majority of mesh testing data is developed from in vivo experimentation on animals—a field which lacks standardization [14, 15]. In an effort to standardize the mesh testing field, our group have begun to explore the possibility of medical device testing in the form of in vitro 3D tissue models. Such models could act as an adjunct to, or preliminary work toward, animal and human trials.

Of the many different attributes that meshes are tested for (tissue integration, immune reaction etc.) mesh shrinkage is perhaps one of the easiest to measure, and simplest to reproduce in vitro. Whilst it is difficult to develop good evidence linking mesh shrinkage to hernia recurrence, it is considered by many to be an important factor determining mesh performance [16] as it can reduce effective mesh overlap. Assessment of mesh shrinkage has been investigated in cohort studies through MRI [17] and novel X-ray methods [18], and linked to increased recurrence rates in some studies [19]. In both animal models and humans, mesh contraction (or shrinkage) is caused by fibroblast-mediated contraction as a result of an inflammatory response to the foreign body [16]. Whilst we cannot yet replicate 3D in vitro immune responses, we can create a 3D model of collagen contraction known as a fibroblast populated collagen matrix (FPCM) that has been established as a model of wound contraction and tissue healing since 1979 [20] and has been used in a host of experiments to examine cell–matrix behaviour [21].

This study has been designed to investigate the potential use of FPCMs for the purposes of testing hernia mesh shrinkage. Specifically, the study seeks to investigate whether hernia meshes can be integrated into FPCMs, and whether contraction of those FPCMs will result in differential mesh shrinkage. This study also seeks to investigate whether this model results in a measurable difference in the release of MMP-9 (a cytokine associated with matrix re-modelling).

## Materials and methods

### Cells and culture techniques

A Human Dermal Fibroblast (HDF) cell line (Thermo Fisher Scientific—Waltham MA, USA) was used for this study (Catalogue number: C0135C). HDFs were grown in Dulbecco’s Modified Eagle’s Medium (DMEM—Sigma-aldrich St Louis, MO, USA), supplemented with 10% foetal bovine serum (FBS) (Thermo Fisher Scientific Waltham MA, USA) and 1% penicillin/streptomycin (Capricorn scientific—Ebsdorfergrund, Germany). Cells for this study were used between passage two and four and cultured in 175cm^2^ (T-175) culture flasks (Thermo Fisher Scientific Waltham MA, USA). Cells were cultured at 37 °C/5% carbon dioxide (CO^2^). Media were changed once every four days until approximately 80% confluence was achieved. HDFs were dissociated from flask bottoms by first washing with phosphate-buffered saline (PBS), then treating with 5 ml of trypsin (0.5%, 1× EDTA; Gibco™) and incubated at 37 °C at 5% CO^2^ for 3–5 min. After successful dissociation was observed under microscopy, 10 ml of culture media were added to flasks to neutralize the effect of trypsin and form the cell stock. Stock was then centrifuged for 5 min at 800G until a pellet was formed. Supernatant was removed and the pellet was resuspended in culture media to provide the required cell density.

### Mesh samples

Five different meshes were used: one polyester ProGrip™ mesh (Medtronic Dublin, Ireland), three undyed PP UNILENE™ meshes (UNISUR Lifecare PVT. LTD., Bangalore, India) and one commercial mosquito net (CarePlus™) (Table 1). Meshes were chosen to provide a breadth of characteristics including material, pore size and weight. Mosquito net was included in the experiment given previous work done to characterize such products [22] and discussion about their use as a frugal alternative for mesh repair [23]. To produce test samples, 1cm^2^ square-shaped pieces were cut from each mesh using a standardized stainless-steel punch (1cm^2^ square punch shape, Oumefar, Kerlife-US Via www.amazon.co.uk).

**Table 1:** All five meshes used for experimentation

Trade name	Material	Weight	Pore size	Filament diameter	Thickness
ProGrip™	Monofilament polyester mesh embedded with resorbable polylactic acid	Lightweight (35g/m^2^)	Macroporous	N/A	N/A
Unilene Mesh™	Monofilament undyed PP non-absorbable surgical mesh	Heavyweight (120 g/m^2^)	Microporous (0.93 mm × 0.65 mm)	0.15 mm	0.65 mm
		Lightweight (35 g/m^2^)	Medium pore (1.31mm x 2mm)	0.15 mm	0.55 mm
		Lightweight (27g/m^2^)	Macroporous (1.48mm × 2.41mm)	0.1 mm	0.39 mm
CarePlus™	Mosquito net (polyester)	N/A	Macroporous	N/A	N/A

Key characteristics of each mesh including pore size and weight are provided (if available)

### Preparation of free-floating FPCMs

**Fig. 1:**
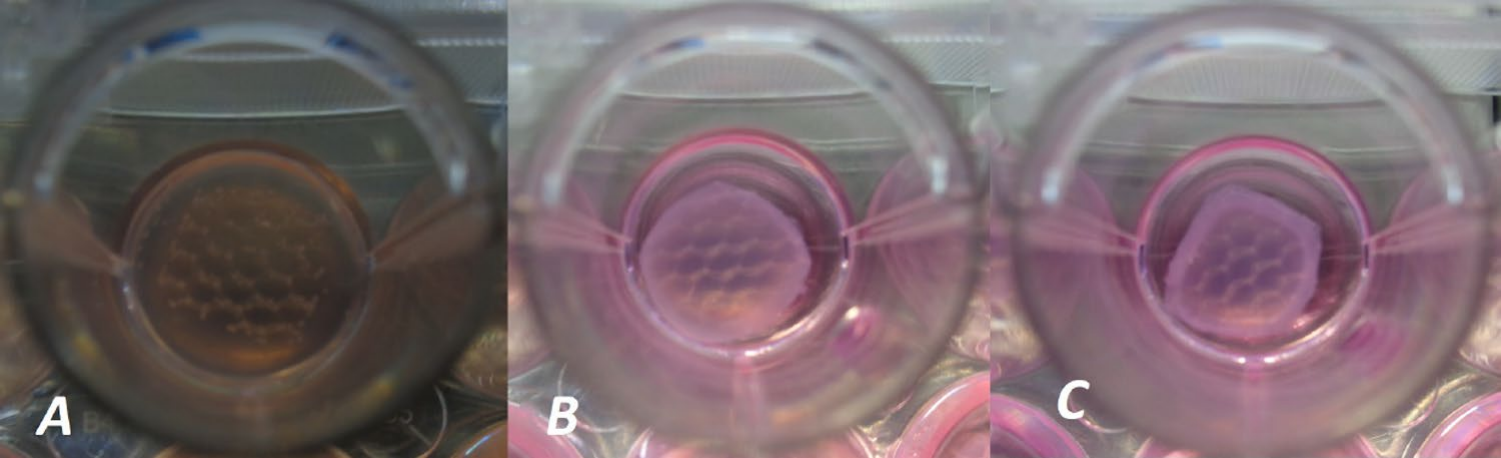
Three photographic images taken of the macroporous mesh integrated within the high cell density FPCM (1.5 × 10^6^). Images **A**, **B** and **C** are taken at 24, 72 and 120 h (days 1, 3 and 5) of culture respectively. The reduction of size in both collagen and mesh can be clearly observed. Note also the visible increase in collagen density—especially at the periphery of the gels

**Fig. 2:**
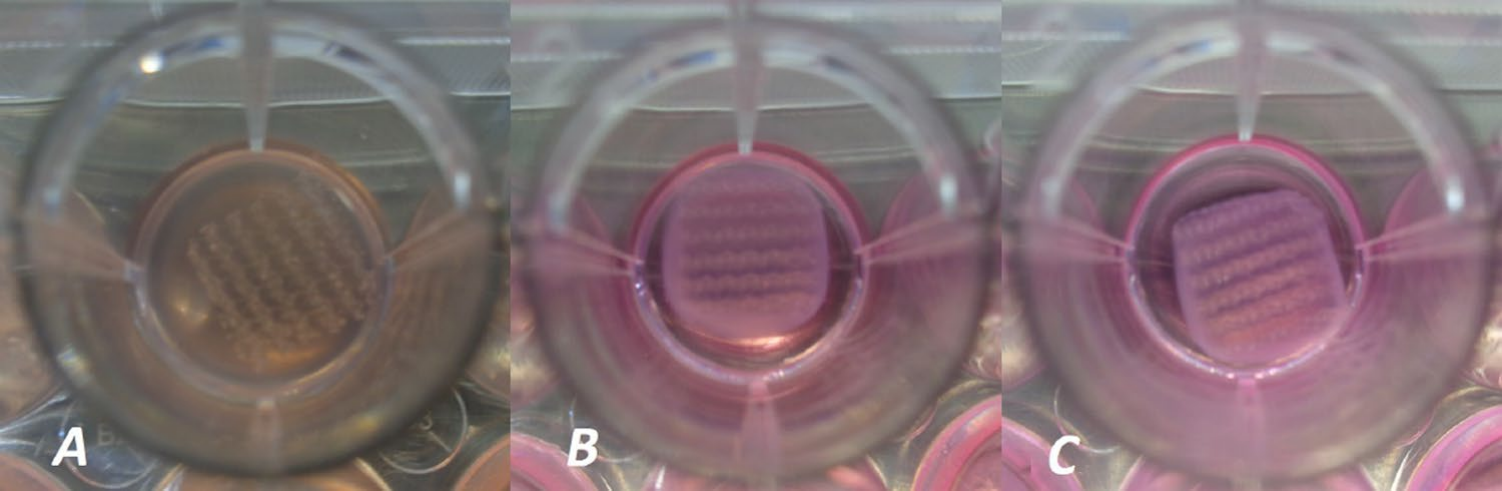
Three photographic images taken of the microporous mesh integrated within the high cell density FPCM (1.5 × 10^6^). Images **A**, **B** and **C** are taken at 24, 72 and 120 h (Days 1, 3 and 5) of culture respectively. The reduction of size in both collagen and mesh are less clearly observed than the macroporous mesh in Fig. 1

FPCMs were prepared on ice to prevent premature setting of collagen. FPCMs were produced as per a published protocol [24]. In brief, the following reagents were combined to the desired volume; 80% type I rat tail collagen (2 mg/ml protein in 0.6% acetic acid: First Link UK Ltd, West Midlands, UK), 10% of 10× concentration of Minimum Essential Medium (10X MEM; Gibco™), 5.8% of neutralizing solution (HEPES Buffer with 1 M Sodium Hydroxide) and finally 4.2% cell stock solution. Six 500 µl FPCMs were cast into the wells of a 24-well plate (Corning™ Costar™—NY, USA) and into five of those were integrated one of five different 1 cm^2^ square-shaped mesh samples. The sixth FPCM served as a non-mesh control. Mesh samples were integrated into FPCMs by first casting the cellularised gel into the well plate, and then pressing the mesh from above into the gel, so that the mesh became fully submerged. Each set of six mesh/FPCM constructs were then repeated at three different cell densities; 0.5, 1 and 1.5 × 10^6^ cells/ml. A single acellular control FPCM was also produced. FPCMS were incubated at 37 °C/5% CO^2^ and allowed to set into a solid gel. 1 ml of culture media were then added to each well and all FPCMs were scored away from the inside of their well perimeter using a sterile needle. This process produced free-floating FPCMs (see Figs. 1a, 2a) which were incubated at 37 °C, 5% CO^2^ for 5 days (120 h). Culture media were changed daily.

### Assessment of collagen contraction and mesh shrinkage

Each collagen construct was photographed at 24 h intervals; images being captured using a digital camera (Cybershot; Sony Tokyo, Japan) and analysed using software package ImageJ™ (National Institutes of Health, Bethesda, MD, USA). This software was used to calculate the area of the upper surface of each FPCM. Each area was measured three times and a mean calculated. Measurements were used to calculate the percentage reduction in area of each FPCM throughout the experiment.

### Assessment of changes to mesh pores

After the 5-day culture period, excess media were removed from each well and constructs were dehydrated using Whatman paper. Collagen constructs were placed back into respective wells of the 24-well plate and light-microscopy images obtained using a digital imaging microscope (EVOS M500, Invitrogen—Waltham MA, USA). Images of pores within the PP Medium Pore, PP macroporous and mosquito net mesh high cell density constructs (1.5 × 10^6^ cells/ml) were captured at 4× magnification. Qualitative observation was made comparing different regions of the mesh and the collagen matrices to examine for any structural differences.

### Fluorescence microscopy

The same samples used for light microscopy (high cell density PP Medium Pore, PP macroporous and mosquito net) were washed with PBS and fixed in 4% paraformaldehyde (PFA) for 30 min. Thin (approx 2 mm) 1 cm long strips were cut from each mesh/FPCM construct and permeabilised in 1 ml of 0.5% Triton X in PBS (Sigma-Aldrich, St Louis, MO, USA) for 30 min. Samples were washed three times in PBS then stained using 200 µl of phalloidin solution (Cambridge Bioscience—Cambridge UK)—incubating samples in the dark at room temperature for 45 min. Following this, one drop of 4′,6-diamidino-2-phenylindole (DAPI) stain solution (Thermo Fisher Scientific—Waltham MA, USA) was added to each sample and incubated in the dark for a further 15 min. Samples were then mounted onto standard microscope slides underneath cover slips. Samples were imaged at 20× using a Zeiss LSM 710 fluorescence microscope (Zeiss—Obercochen, Germany). Images from at least two different locations of each construct were taken to view the distribution of the cells within the pore of the mesh and the edge of the mesh/collagen matrix.

### Matrix metaloproteinase-9 (MMP-9) ELISA

Culture media from contracting FPCMs was collected on every day of culture for each mesh and stored at −80 °C before being used to measure MMP-9 release using an enzyme-linked immunosorbent (ELISA) assay (*Legend Max*, BioLegend—San Diego, CA, USA). Only samples from day 2 and day 4 were used to measure MMP-9 release. Experimental procedures were followed using manufacturer’s instructions. In brief, the assay was performed in a 96 well plate pre-coated with a mouse monoclonal anti-human MMP-9 antibody. The human MMP standard was prepared by diluting 80 µL of the stock solution in 420 µL of assay buffer, and six, twofold dilutions were performed. Media samples from FPCMs were also prepared at five different twofold dilutions. All wells were incubated at room temperature for 2 h. After incubation, plates were washed thoroughly at least four times with 1X wash buffer before human MMP-9 antibody detection solution was added into each individual well and incubated further for 1 h at room temperature, followed by another series of washes. After this, Avidin-HRP peroxidase solution was added to each well and incubated for 30 min at room temperature before the final series of washes were performed. Bound HRP is then detected by adding a substrate solution and wells containing human MMP-9 changed to blue in colour with the intensity directly proportional to MMP-9 concentration. The reaction was stopped, and the plate was scanned for absorbance at 450 nm by a microplate reader (Infinite M Plex, tecan—Mannedorf, Switzerland).

## Results

### Collagen contraction

**Fig. 3:**
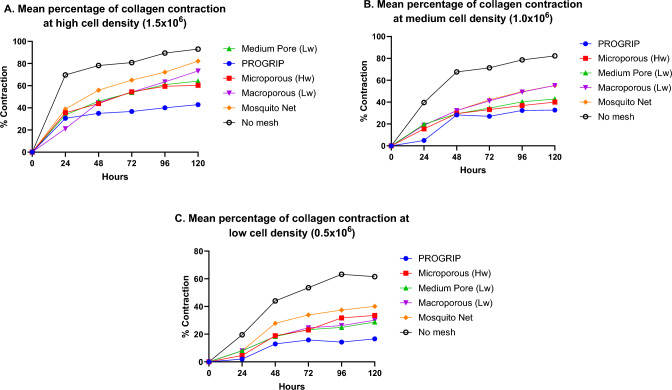
Graphs showing the average percentage of collagen contraction over 120 h at three different cell densities. Hw = heavyweight, Lw = lightweight

All cellular FPCMs demonstrated the ability to contract, and as expected with well-plate contraction assays [25], higher cell densities produced greater levels of collagen contraction. At the highest cell density, there was a rapid increase in collagen contraction with all mesh types up to 48–72 h followed by a gradual plateau thereafter. Collagen contraction was greatest in high cellular density gels integrated with mosquito net (82%) and PP macroporous mesh (73%) at 120 h, followed by PP medium pore mesh (64%), PP heavyweight (60%) and PET mesh (43%). At 120 h, the high-density cellular controls demonstrated capacity to contract by 92% in high cellular density gels. At medium and low cellular densities, collagen contraction was slower, peaking at 120 h to 78% in the medium cellular density gels and by 54% in the lowest cellular density gels (Fig. 3).

### Mesh shrinkage

**Fig. 4:**
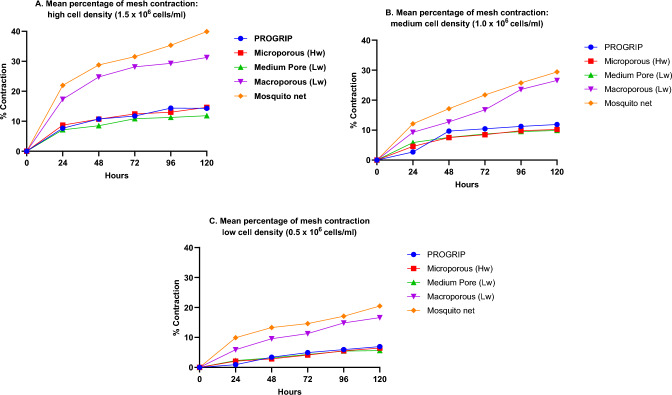
Graphs depicting the average percentage of mesh contraction over 120 h period at three different cell densities. Hw = heavyweight, Lw = lightweight

All meshes reduced in size throughout the 5 days of culture; however, rates of mesh shrinkage varied dependent upon both cell density and mesh type. Figures 1 and 2 demonstrate the relative shrinkage of different meshes over 5 days. Figure 4 summarizes the percentage of mesh shrinkage over 5 days (120 h) for different meshes at different cell densities. The plots provide mean values from 3 measurements of the same mesh. After 24 h, shrinkage was most prominent in the mosquito net seeded at the highest density (22%), followed by the PP macroporous mesh (17%). These two meshes remained the most contractable materials throughout all timepoints and all cell densities. In each of the three graphs, a similar pattern can be observed, where meshes appear to fall into two broad categories—in which the mosquito net and the PP macroporous mesh were found to contract a greater amount than other products. This pattern is most clearly observed in the FPCMs with the highest cell density.

### Changes to pore shape and size

**Fig. 5:**
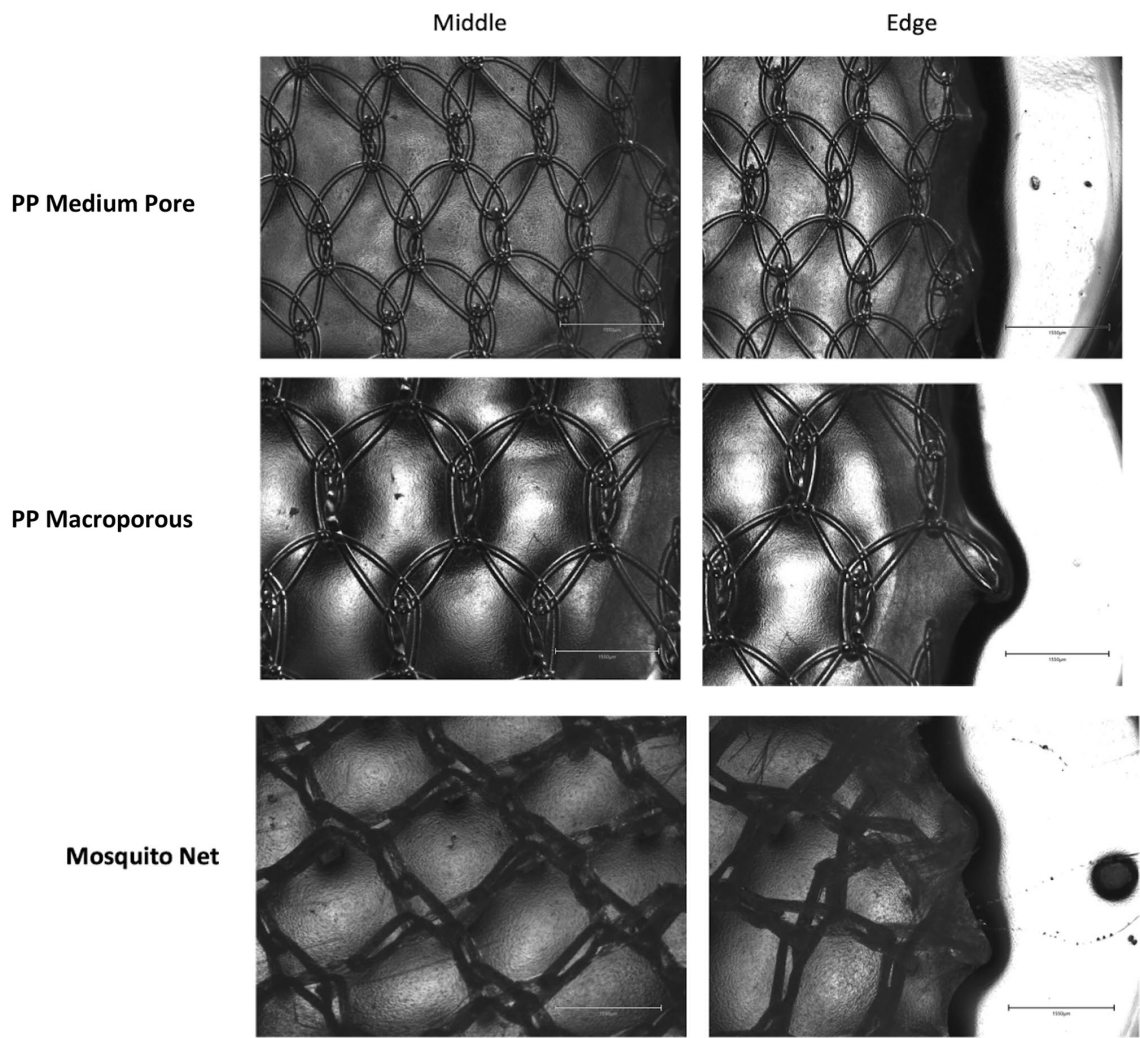
Light microscopy images of 3 separate mesh products (PP medium pore, PP macroporous and mosquito net) within FPCMs after 5 days (120 h) of culture. Images are provided of both the central portion of the mesh (left sides images) and those on the edge of the mesh (right sided images). Images are taken with a 4× objective, scale bars 1550 µm

After culture, FPCMs were dehydrated and imaged under 4× magnification. As seen in Fig. 5, there was no observable difference between the pores in the middle of the mesh and the edge of the mesh in both the PP medium pore mesh and the PP macroporous mesh. However, the edges of the mosquito net suffered significant crumpling around its edges.

### Cell distribution

**Fig. 6:**
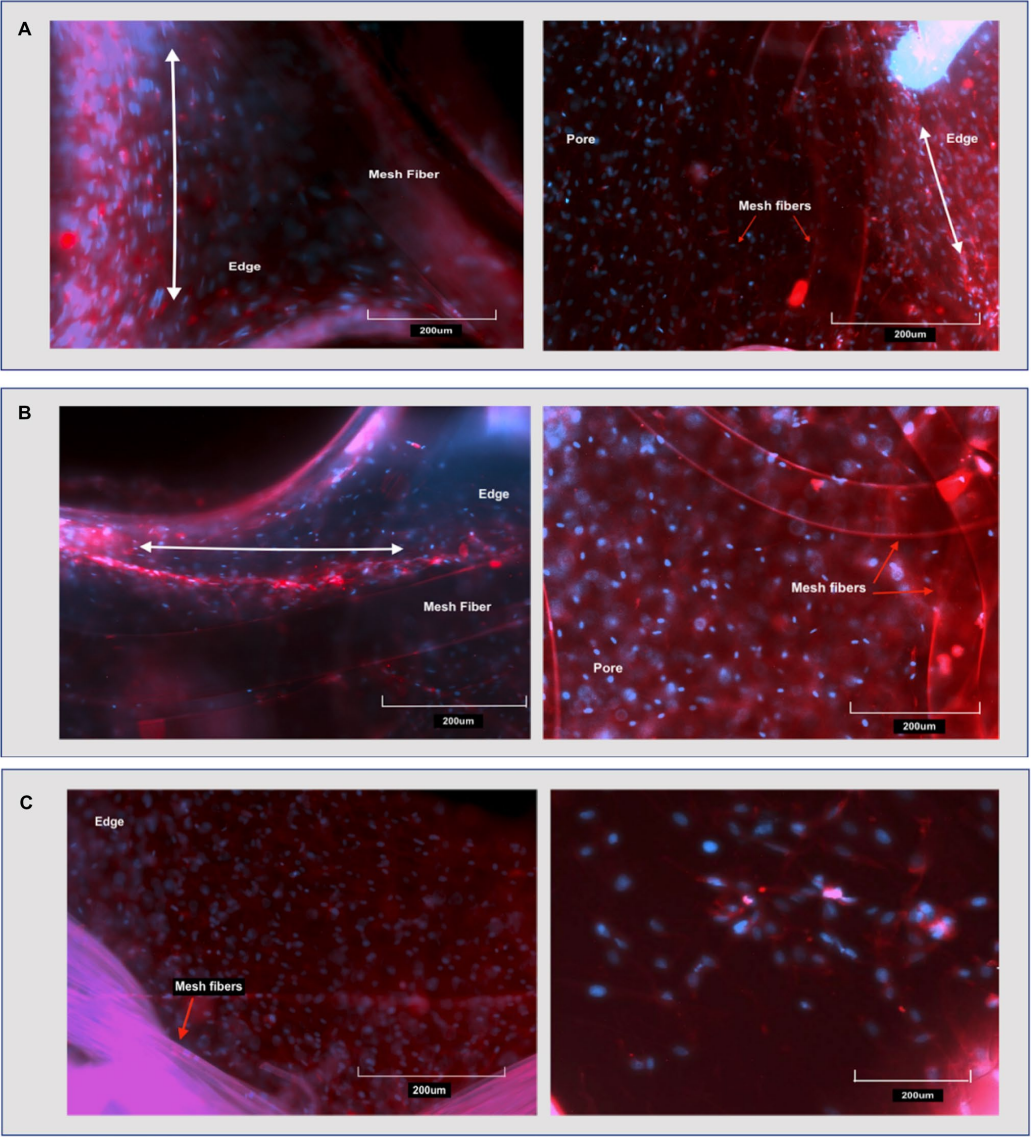
Fluorescence microscopy images showing Cell distribution at different regions (edge, pore and mesh fibre regions) of the collagen constructs with different integrated meshes at the highest cell density (1.5 × 10^6^ cells/ml). **a** PP medium pore mesh. **b** PP macroporous mesh. **c** mosquito net. Right sided images are from the central mesh pores, whilst left sided images are from the edge of the mesh. White double headed arrows indicate cellular alignment. Image objective is 20×, and scale bars are 200 µm

After 5 days of culture, constructs with the highest rates of contraction (i.e. the high cell density mosquito net and the PP macroporous mesh) were fixed with PFA and stained with phalloidin and DAPI. The PP medium pore mesh was also stained for comparison. Fluorescence microscopy images were taken both within the pores of the mesh and the edge of the mesh. Imaging showed an abundance of healthy elongated HDFs within the pores of all three meshes (Fig. 6a–c) and a higher concentration of cells at the edge region. Figure 6a, andb demonstrates not only increased cell density but also cellular alignment at the edge sections of both meshes. Cellular alignment is demonstrated not only by the phalloidin-stained actin filaments, but also the DAPI-stained nuclei that can be seen forming ovoid shapes that lie in the same direction. Cells within the pores of the mesh do not exhibit this increased cell density nor cellular alignment. Cell attachment to mesh fibres was also observed in all three meshes.

#### MMP-9 ELISA

The MMP-9 assay (data not shown) revealed no observable significant differences in MMP-9 release between different types of mesh at both 48 and 96 h of culture. MMP-9 levels were negligible on both days so accurate quantification could not be performed. It is likely such low MMP-9 levels were secondary to daily media changes as well as dilution of the media through matrix contraction. Despite these low readings for our test samples, control samples of human MMP-9 were detected at a range of concentrations—establishing a working assay.

## Discussion

This study was designed to explore the development of a 3D in vitro tissue model that can compare rates of shrinkage between different commercial hernia meshes. Although previous groups have studied in vivo mesh shrinkage in great detail [26], this study is the first to model the process in vitro. Whilst the results of this study cannot be said to reflect in vivo conditions, and, therefore, must be treated with caution, we believe that the work has made a number of important steps toward the development of a functioning model for in vitro mesh testing. First, it has established that different commercial hernia meshes of different materials can be successfully integrated into cellular collagen matrices. Not only do the cells within these matrices survive, but they continue to be functionally active in terms of matrix contraction. Second, this model has established its ability to initiate contraction (or shrinkage) of commercial hernia meshes and for that shrinkage to be appreciable and measurable macroscopically. Finally, this work strongly suggests that our model can cause shrinkage of different meshes at varying rates, and that these differences are consistent across different cell densities.

Graphs across Fig. 3 show consistently that the ProGrip™ mesh as well as the heavyweight PP and lightweight medium pore PP meshes all shrank at similar rates. In contrast, the PP macroporous mesh and mosquito net shrank both more quickly and to a greater extent. Whilst each mesh has a number of different structural characteristics, this shrinkage within this study appears to be related to the relative “stiffness” or malleability of each product. Other authors have discussed how both mesh material and pore size may affect mesh shrinkage [16, 27]. Within their literature review, Brown et al. made strong assertions that not only do different mesh *materials* shrink at different rates, but also smaller pore meshes tend to shrink more due to increased inflammation and scar plate formation [27]. The formation of an inflammatory reaction is a key component of mesh reaction and is therefore something that would create a more biomimetic model if introduced.

We believe that the use of 3D tissue models provides a novel and biomimetic technique for mesh testing which provides distinct advantages over other in vitro testing techniques. Using cell culture techniques to test hernia meshes in vitro has been explored a number of times in the literature. Similar to our work, Fehér et al. [28] used HDF cells to test hernia meshes by seeding them directly onto different products. Whilst the study was able to stain the cells and assess their morphology, cellular function was not assessed. Another study by Giuntoli et al. [29] again used HDF cells seeded directly onto mesh and assessed cell proliferation by measuring collective metabolism over time. Other interesting studies have used human omental cells to undertake similar work—looking to explore the potential interactions with mesh placed intraabdominally [30]. With such techniques, one obvious critique is that when cells are seeded directly onto mesh (in 2D), some cells will not adhere to mesh, falling between fibres to the bottom of the well plate. Data, therefore, will be affected by the relative surface area of the mesh being tested. Using a 3D tissue model ensures that not only are cells suspended in a more biomimetic collagen matrix, but also the activity of all cells within the matrix around the mesh can be assessed, as well as those adherent to mesh.

The advantage of modelling tissues in 3D is further exemplified by this study’s finding of increased cellular density and alignment around the edges of certain mesh products. This alignment and increased density was clearly observed at the edge of two PP meshes (Fig. 5a, b) but was absent at the edge of the mosquito net (5c). Such density and alignment was also absent within the pores of each mesh. It is known that cell proliferation and alignment can be affected in vitro by the application of mechanical forces [31–34]. Given that contraction of the FPCMs was reduced (or resisted) by the presence of hernia meshes (see Fig. 3), it stands to reason that stiffer, less contractable materials would create a greater resistance to matrix contraction at the edge of the mesh. It could be hypothesised that it is this contraction of collagen against a stiff biomaterial that leads to increased cell alignment. Whilst this observation requires further validation and quantification with much larger studies, it could prove important for modelling the behaviour of native tissue fibroblasts at the edge of fixed mesh products.

As well as examining overall shrinkage, meshes were examined to assess the potential shrinkage or change of shape to individual pores. There were, however, no discernible differences between “edge” and “central” pores in either the medium pore or macroporous PP meshes. Microscopy did, however, reveal significant lateral crumpling of pores toward the edge of the mosquito net—likely due to its increased pliability and multifilament structure. It is likely that any overall change to mesh size/structure is occurring due to a vertical folding within the mesh, which would not be measurable once meshes were removed from culture and dehydrated. More detailed imaging methods such as in-culture microscopy may help future experiments analyse changes within pores during culture.

This study also set out to examine how mesh products may effect MMP-9 release. MMPs are vital components of the wound healing process, MMP-9 in particular is recognized as a regulator of contractile activity driven by fibroblasts [35, 36]. It is possible therefore that MMP-9 release may be altered by either different mesh products, rates of contraction or culture time. Results from the MMP-9 ELISA assay revealed insufficient MMP-9 release to quantify and/or make viable conclusions. This lack of MMP-9 could be secondary to dilution caused by two factors. First, as FPCMs contract, they release fluid into surrounding media—diluting its MMP concentration. Second, media were collected and changed every day of culture, only giving 24 h for MMP-9 to collect within the media. It is likely, therefore, that our model in its current design requires further optimization before such enzymatic assays can be useful.

Clearly this work is not without its limitations. The photographic technique used for measuring mesh and collagen shrinkage could be improved as the depth of the well plate and its perception by a 2D image may affect accuracy. Steps were taken to minimise the effects of this, including making measurements relative to a fixed size shape (the well plate edge) and taking multiple measurements. Regardless, the rate of mesh shrinkage over 5 days cannot be used to infer similar behaviour in animal studies. The model in this study is produced from human dermal fibroblasts—rather than cells from the abdominal wall. Our group has now, however, managed to isolate and culture human fascial (rectus sheath) fibroblasts [37] which will be integrated into future models. In vitro models such as ours could be further optimized by the introduction of an immune component—closer mimicking the human healing process. Future work with this model could also seek to better understand the cytotoxicity of different mesh products by using live/dead or metabolic assays. Further work should also be done to optimize the measurement of MMP release from FPCMs; exploration of techniques such as zymography may help to provide more reliable data. Once such in vitro models have been optimized and made sufficiently biomimetic, their performance would be best validated by comparison either with human studies, or standardized animal models.

## Conclusion

We believe that this study describes the first work to explore the use of 3D tissue models to test hernia mesh in vitro. Whilst results cannot be compared to those of in vivo studies, it acts as a promising and novel proof of concept for future work. Future experiments hold the potential to expand upon this work and develop a reproduceable standardized technique for future mesh testing—either as an adjunct to, or precursor for, other animal/human studies.

## Data Availability

This work only invlved the use of small data sets - the majority of which are published here. Any further data is availble upon request of the corresponding author.
